# Repetitive transcranial magnetic stimulation (rTMS) in alcohol dependence: study protocol of a randomized controlled clinical trial of efficacy and working mechanisms

**DOI:** 10.1186/s12888-018-1759-y

**Published:** 2018-06-04

**Authors:** Renée S. Schluter, Ruth J. van Holst, Anna E. Goudriaan

**Affiliations:** 1 Department of Psychiatry and Amsterdam Institute for Addiction Research, Academic Medical Center, University of Amsterdam, Amsterdam, The Netherlands; 2 Donders Institute for Cognition, Brain and Behaviour, Radboud University, Kapittelweg 29, 6525 EN Nijmegen, Gelderland The Netherlands; 3 Arkin Mental Health Care, Klaprozenweg 111, 1033 NN Amsterdam, Noord-Holland The Netherlands

**Keywords:** Alcohol dependence, HF-rTMS, Abstinence, Craving, Neurocognitive, Neurobiological

## Abstract

**Background:**

High frequency repetitive transcranial magnetic stimulation (HF-rTMS) has gained interest as a neuromodulation treatment technique for alcohol dependence. Single sessions of HF-rTMS have consistently shown to decrease craving for substances. However, the results of randomized controlled clinical trials investigating the effect of multiple HF-rTMS sessions in alcohol dependence on abstinence rates and craving are inconsistent. Furthermore, they lack information on the effect of HF-rTMS on cognition and brain functioning.

**Methods:**

A single center, single blind, randomized controlled trial with 80 abstinent alcohol dependent subjects in treatment randomized (1:1) to either treatment as usual (TAU) plus ten sessions of active HF-rTMS or TAU plus 10 sessions of placebo/ sham HF-rTMS will be performed. The effects of ten HF-rTMS sessions on craving and neurocognitive functions are obtained. In addition a subset of participants will undergo an MR scanning session before the first and after the last HF-rTMS session in order to investigate the effect of ten HF-rTMS sessions on brain functioning. The primary outcome is the continued abstinence rate after the add-on HF-rTMS treatment.

**Discussion:**

This study uses a randomized controlled trial to examine the clinical, neurocognitive and brain functioning effects of ten add-on HF-rTMS sessions in alcohol dependent individuals in treatment. If the add-on treatment is effective, this may add to the evidence needed for approval of this additional treatment method for alcohol dependence by regulatory authorities.

**Trial registration:**

The Netherlands National Trial Register (NTR), NTR5291, 6-July-2015.

## Background

Substance dependence is characterized by drug seeking and drug use which persists despite negative social and health consequences [1]. In the Netherlands approximately 4–5% of the population is suffering from an alcohol use disorder [2]. In 2014 more than 30.000 individuals were registered at addiction treatment centers in the Netherlands with alcohol as primary substance of abuse [3]. Currently only psychosocial and pharmacological treatments are available for alcohol use disorders. However, these treatments are only moderately effective and more than 50% of all treated patients relapses within one year [4]. In an attempt to improve the treatment of substance dependence, non-invasive neuromodulation has gained attention as a new potential treatment option [5, 6].

High-frequency (HF) repetitive transcranial magnetic stimulation (rTMS) [7] is one of several types of neuromodulation techniques. With this method, a magnetic field penetrates through the skull which can inhibit or activate neurons in the cortex. This magnetic field originates from a coil wherein an alternating electric current is running. The alternating current induces high intensity magnetic pulses that pass the skull and generate an electric current in the neural tissue. By using this technique the activity of the targeted cortical area is manipulated [5, 8]. HF-rTMS became a popular investigational treatment tool for several psychiatric disorders because of its non-invasiveness, tolerability and safety [9].

Individuals suffering from alcohol dependence often experience an intense and abnormal desire for alcohol, also known as craving [10]. Furthermore it is known that alcohol dependent individuals show impaired executive functions, such as diminished cognitive control, cognitive flexibility and working memory [11, 12]. It is believed that perceived craving combined with reduced cognitive control leads to problems in managing craving and consequently relapse [1].

On a neurobiological level craving is associated with heightened striatal activity related to addiction-relevant stimuli, whereas diminished cognitive control is associated with decreased prefrontal activity [13]. One of the areas involved in cognitive control is the dorsolateral prefrontal cortex (DLPFC) [14]. By stimulating the DLPFC with a high-frequency (10 Hz) protocol, the neural activity of this area is enhanced [15] thereby improving cognitive control [16, 17]. Furthermore, it is known that the prefrontal cortex has abundant connections with the striatum [18]. Indeed neuromodulation of the DLPFC has shown to induce changes in neurotransmitter concentrations in the striatum [19] and can decrease feelings of craving with a medium effect size [d = 0.48] (Jansen et al., 2013). The DLPFC therefore seems an excellent target area for treating alcohol dependence because it could enhance cognitive control functions while also influencing striatal functioning and reducing feelings of craving [5]. However, results of clinical trials investigating the effect of *multiple* HF-rTMS sessions on craving are very scarce and inconsistent, with one study stimulating the right DLPFC that reported reduced craving [20] and one study stimulating the left DLPFC that failed finding an effect on craving [21]. Furthermore no studies thus far investigated the effect of multiple HF-rTMS sessions on abstinence rates after a longer period of time.

Altogether the effect of multiple HF-rTMS sessions on abstinence rates, perceived craving and the neurocognitive and neurobiological working mechanisms are poorly investigated (see also, [22]). The current study aims to elucidate the effect of *multiple* sessions of HF-rTMS on abstinence, craving, cognition and brain functioning. We will conduct a single center, single blind, randomized controlled trial with 80 abstinent alcohol dependent subjects in treatment randomized (1:1) to either treatment as usual (TAU) plus 10 sessions of active HF-rTMS or TAU plus 10 sessions of placebo/ sham HF-rTMS. The effect of active versus sham HF-rTMS treatment will be investigated on measures of abstinence, craving, cognition and brain functioning. We expect higher abstinence rates and decreased perceived craving together with improved functioning on the neurocognitive tasks and brain functioning measures in the active HF-rTMS treated group compared with the sham HF-rTMS treated group.

### Aims of the study

This study aims to investigate the efficacy and working mechanisms of 10 add-on HF-rTMS sessions in a treatment-as-usual setup of alcohol dependence.

- Primary research question:
  - What is the effect of ten sessions of active HF-rTMS on abstinence rates in alcohol dependent individuals in treatment, compared with ten sessions of sham HF-rTMS?
- Secondary research questions:
  - What is the effect of 10 sessions of active add-on HF-rTMS treatment on total amount of alcohol consumed after treatment in alcohol dependent individuals, compared with 10 sessions of sham HF-rTMS?
  - What is the effect of 10 sessions of active add-on HF-rTMS treatment on days until first relapse after treatment in alcohol dependent individuals, compared with 10 sessions of sham HF-rTMS?
  - What is the effect of 10 sessions of active HF-rTMS on perceived craving levels in alcohol dependent individuals in treatment, compared with 10 sessions of sham HF-rTMS?
- Additional research questions:
  - What is the effect of 10 sessions of active HF-rTMS on performance on neurocognitive tasks measuring impulsivity, approach avoidance, spatial working memory and compulsivity in alcohol dependent individuals in treatment, compared with 10 sessions of sham HF-rTMS?
  - What is the effect of 10 sessions of active HF-rTMS on brain functioning measures related to cognitive control and craving in alcohol dependent individuals in treatment, compared with 10 sessions of sham HF-rTMS?

## Methods/ design

### Study design

The effectiveness of the HF-rTMS add-on treatment will be tested in a parallel, single center, single blind trial in abstinent alcohol dependent subjects, randomized (1:1) to either treatment as usual (TAU) plus 10 sessions of active HF-rTMS or TAU plus 10 sessions of sham HF-rTMS. A subset of the participants will undergo a magnetic resonance imaging (MRI) scan prior to the first and after the last stimulation session in order to investigate the effects of HF-rTMS on brain functioning (referred to as neuroimaging study part).

### Ethical considerations

This study is approved by the Medical Ethical Committee of the Academic Medical Centre Amsterdam (2015_064) and is registered in The Netherlands Trial Register (NTR) with trial number 5291. Written informed consent is obtained before screening for in and exclusion criteria takes place.

### In- and exclusion criteria

All participants will be recruited when they are three to four weeks abstinent, and are recruited from the Jellinek Addiction Treatment Centre in Amsterdam, The Netherlands. Inclusion criteria are a recent (less than four months after detoxification) DSM-IV diagnosis of alcohol dependence and age between 20 and 65. Exclusion criteria are (1) insufficient knowledge of the Dutch language, (2) Montreal Cognitive Assessment (MOCA) score below 10, (3) current DSM-IV diagnosis of depression, schizophrenia or another psychotic disorder, (4) current recreational drug use, (5) rTMS contraindications (such as a history of epileptic seizures, metal implants near the head, use of imipramine, amitriptyline, doxepine, nortriptyline, maprotiline, chlorpromazine, clozapine, foscarnet, ganciclovir, ritonavir, amphetamines [15]), and if applicable (for the neuroimaging study part) (6) MRI contraindications (such as metal implants or claustrophobia).

### Intervention

The intervention exists of 10 HF-rTMS sessions of the right DLPFC (rDLPFC) on 10 consecutive workdays. The HF-rTMS parameters of the active intervention are 60 10 Hz trains of five seconds at 110% of the motor threshold [23]. The coil will be oriented over the rDLPFC with a horizontal angle of 45° relative to the nasion-inion midline [24]. For the sham stimulation the stimulator will be set at the same settings, but the coil will be tilted 90° relative to the skull [23]. The rDLPFC will be located at position F4 using the International 10–20 EEG system [25]. During the stimulation participants are situated on a comfortable chair with extra neck support. All sessions are applied at the Jellinek addiction treatment centre in Amsterdam. One stimulation session takes approximately 20–30 min.

The motor threshold will be determined at rest before the first and sixth stimulation session, using single pulse TMS in combination with Motor Evoked Potentials. The muscular (left abductor pollicis brevis) response will be measured by visually observing a thumb muscular abduction. Stimulus intensity will be adjusted until there is an abduction in five out of ten trials. For the rTMS sessions we will use a 70 mm double air film coil (Magstim Co., UK) and a Magstim Rapid^2^ stimulator (Magstim Co., UK). The intervention will be applied by an rTMS trained researcher. The training exists of brain stimulation courses, practical rTMS tutorials, hands on training and first aid and emergency response training. During the stimulation session a predefined protocol will be executed.

The HF-rTMS intervention will be added to the treatment as usual (TAU) provided by the Jellinek addiction treatment centre. This treatment consists of an intensive 3–5 days per week program with group sessions of cognitive behavioural therapy (CBT), emotion regulation training, and motivational enhancement therapy. Besides these sessions, every participant has individual sessions with a psychologist and a mentor every week. In the session with the psychologist, comorbidities and other problems of the patients that occur during treatment are discussed. During the mentor session supportive CBT focussing on remaining abstinent is given. Finally, some patients receive pharmacotherapy.

### Procedure and data collection

#### Inclusion procedure

The participants will start their TAU at a clinical or day detoxification unit where they stay for 7–10 days, and continue their treatment during an intensive 3–5 days treatment of at least three weeks. During the beginning of their treatment participants are informed of the study by the researcher. Individuals who are interested in participation are invited for an appointment to provide them with more information. If they are interested, informed consent is signed and the participant is screened for inclusion and exclusion criteria. If a patient meets all inclusion criteria and none of the exclusion criteria he/she will be included in the study by the researcher. After inclusion the patients can indicate whether they also want to participate in the neuroimaging study part. In that case a subsequent MR screening is performed. In order to assure concealed randomization, participants are assigned to the sham or active stimulation group after inclusion, based on the stratification factors anti-craving medication (yes / no) and age (20–40 / 41–65) using variable block sizes (4, 6 and 8) of the randomisation module implemented in the data management system Castor EDC (Castor Electronic Data Capture, Ciwit BV, Amsterdam, The Netherlands, 2016). After randomization participants start with the research procedure described below.

#### Overview of study

**Fig. 1:**
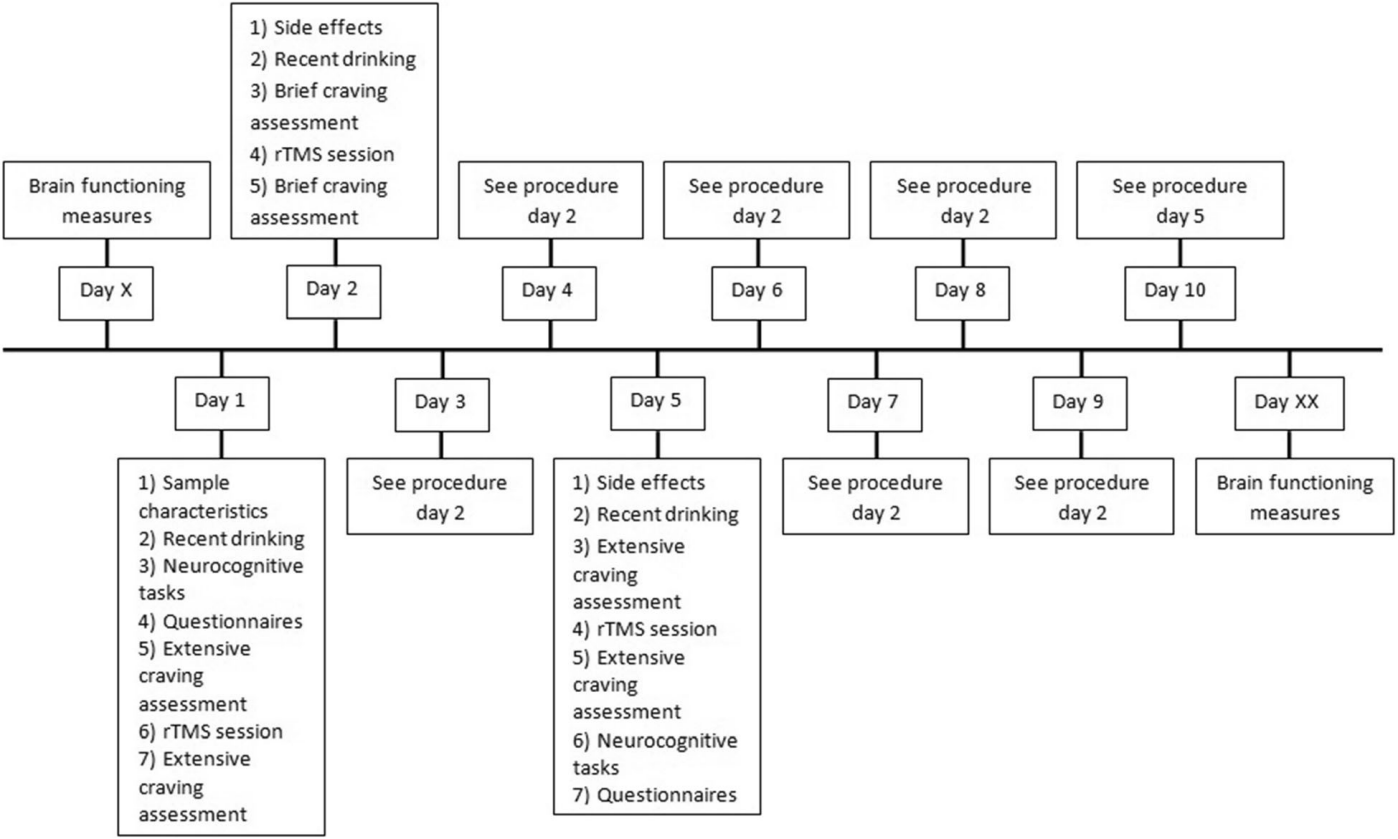
Overview of the measures taken on different test days. Sample characteristics (age, handedness, educational level, medication use, WAIS digit span, NLV, MINI, substance use during life, alcohol use history), neurocognitive tasks (GNGT, AAT, DDT, SWMT, SST, IDED), questionnaires (AUDIT, AASE, BDI, BIS, UPPS, BIS/BAS, STAISTATE, PANAS), extensive craving assessment (AUQ, OCDS, VAS), brief craving assessment (VAS). All these measures will be explained in more detail in the *outcomes and instruments* section. Symbols: X = week before first rTMS session, XX = week after last rTMS session

For an overview of instruments, order of assessment and moment of assessment see Fig. 1 and Table 1. The first test day takes approximately 4 h, the fifth and tenth test day take three hours and all the other assessments take 45 min. After three, six and 12 m a 30-min telephone follow-up interview will be held.

**Table 1:** Overview of measurement instruments and moment of assessment during study. Symbols: X = MR session week before first rTMS session, XX = MR session week after last rTMS session, XXX = three months after last rTMS session, XXXX = six months after last rTMS session, XXXXX = twelve months after last rTMS session

Test day	X	1	2	3	4	5	6	7	8	9	10	XX	XXX	XXXX	XXXXX
WAIS		•													
NLV		•													
MATE-Q		•													
MINI		•													
Alcohol use history		•													
TLFB													•	•	•
VAS		•	•	•	•	•	•	•	•	•	•		•	•	•
AUQ		•				•					•		•	•	•
OCDS		•				•					•		•	•	•
DDT		•				•					•				
Go/No-go		•				•					•				
SST		•				•					•				
AAT		•				•					•				
SWMT		•				•					•				
IDED		•				•					•				
Urine test	•											•			
Cue reactivity	•											•			
Stroop	•											•			
MIDT	•											•			
Resting state	•											•			
ASL	•											•			
AASE		•				•					•		•	•	•
AUDIT		•													•
BDI		•				•					•				
BIS/BAS		•				•					•				
BIS		•				•					•				
UPPS		•				•					•				
STAI/STATE		•				•					•				
PANAS		•				•					•				
Side effects			•	•	•	•	•	•	•	•	•				
Alcohol use study		•	•	•	•	•	•	•	•	•	•				

##### Neuroimaging study part

The outcome measures concerning brain functioning will be obtained in the week before the first stimulation session (i.e. to measure baseline brain functioning) and in the week after the last stimulation session (i.e. to measure the effect of HF-rTMS). Before the participants enter the scanner they perform a urine alcohol and drug screening and practice the tasks that will be conducted in the MRI scanner.

### Outcomes and instruments

#### Sample characteristics

General patient characteristics such as age, handedness, educational level and use of medication will be assessed. Furthermore the digit span of the Wechsler Adult Intelligence Scale (WAIS) will be used to determine working memory capacity [26]. In addition the Dutch Version of the Adult Reading Test (NLV) will be used to assess premorbid intellectual functioning [27]. The Mini International Neuropsychiatric Interview (MINI) [28] will be used to determine whether the participant has any psychiatric DSM-IV diagnoses. The substance use during life questionnaire from the measurement in the addictions for triage and evaluation (MATE) [29] will be used to assess lifetime drug use. For a comprehensive assessment of the alcohol use history an adapted version of the life time drinking history [30] will be used.

#### Primary outcome measure

The primary outcome of the study will be the abstinence rate after the add-on HF-rTMS treatment (in line with the guidelines of the European Medicines Agency [31]). This will be defined as the number of abstinent days in the 180 days after the last stimulation session measured using the Time Line Follow Back (TLFB) [32] at six months follow up.

#### Secondary outcome measures

The secondary outcome measures of the study will be described below.

- Total alcohol consumption after the add-on HF-rTMS treatment. This amount (g) of alcohol will be calculated using the TLFB at three months and six months follow-up.
- Days until the first relapse after the add-on HF-rTMS treatment, wherein relapse is defined as a heavy drinking day. This holds more than 60 g alcohol per day for men and more than 40 g alcohol per day for women [31, 33]. This measure will be assessed using the TLFB at three months follow up and six months follow-up. Individuals who do not relapse will receive the highest score of 90 days.
- Change in craving levels after the add-on HF-rTMS treatment will be measured using the Alcohol Urge Questionnaire (AUQ) [34] assessed after the last session of the add-on HF-rTMS treatment, at three and six months follow-up.

#### Additional clinical outcome measures

##### Explorative alcohol use parameters

The effect of HF-rTMS on alcohol consumption or abstinence will be assessed using the following explorative outcome measures.

- Full abstinence rate after the add- on HF-rTMS treatment, defined as the number of participants who did not consume any alcohol after the HF-rTMS treatment. This will be assessed using the TLFB at six months follow-up.
- Treatment success based on drinking status at 12 m follow up. The drinking status will be subdivided into the following categories: abstinence (defined as no use of alcohol), non-excessive drinking (defined as 21 drinking days per 30 days, with a maximum of 4 glasses per day) and excessive drinking (more than 21 drinking days per 30 days and more than 4 glasses per day) [4]. Participants will be assigned to one of the categories based on the last 30 days of the 12 m follow up TLFB assessment. The categories abstinence and non-excessive drinking will be defined as successful treatment.

##### Additional craving assessment

The effect of HF-rTMS on craving will also be assessed using the short (5-item) version of the Obsessive Compulsive Drinking Scale (OCDS) [35]. This questionnaire will be assessed prior to the first, and after the fifth and tenth stimulation session and at the 3, 6 and 12 months follow-up.

To assess the acute effect of HF-rTMS on craving two 100 mm visual analogue scale (VAS) ranging for 0 (not at all) to 100 (very much) will be assessed prior and after every stimulation session. Participants will be asked to indicate (1) their current desire to consume alcohol: “How much do you feel like drinking alcohol right now?” and (2) their current urge to drink alcohol: “How strong is your urge to drink alcohol right now?” by drawing a line on a VAS scale.

##### Questionnaires

Questionnaires that will be filled in during the study are:

- Alcohol consumption burden measured with the Alcohol Use Disorders Identification Test (AUDIT) [36].
- Confidence in abstaining from drinking as measured with the Alcohol Abstinence Self Efficacy (AASE) [37].
- Depressive symptoms as measured with The Beck Depression Inventory (BDI) [38].
- Impulsive behavior will be assessed using Barratt Impulsivity Scale-11 (BIS) [39], UPPS (urgency, premeditation, perseverance, sensation seeking) [40] and Behavioral inhibition and Behavioral Activation systems (BIS/BAS) [41] questionnaires.
- Current state anxiety as measured with the state trait anxiety inventory (STAI STATE) [42].
- Experience of positive and/ or negative affect measured with the Positive And Negative Affect Scale (PANAS) [43].

##### Supplementary measures

In order to get an overview of the drinking behavior during the testing period (variable defined as: alcohol use study) participants are asked before every HF-rTMS session “whether (1) he/she consumed alcohol in the last 24 hours and if so (2) what kind of alcohol, (3) the alcohol percentage and (4) the amount consumed”. Furthermore participants are asked whether they were suffering from any side effects after the previous HF-rTMS session. The reported side effects will be listed.

#### Neurocognitive measures

Despite the role of the prefrontal cortex in higher cognitive processes [44], the effect of HF-rTMS over the rDLPFC on neurocognitive measures in alcohol dependent individuals is poorly investigated (see also [22]). Therefore the effect of active HF-rTMS compared with sham HF-rTMS on several neurocognitive tasks will be assessed before the first, after the fifth and tenth HF-rTMS session. Neurocognitive tasks will focus on cognitive control and other relevant processes such as approach behavior and working memory. Participants will be situated in front of a finger operated touch screen tablet (Hewlett-Packard; Windows 8.1) on which the tasks will be conducted.

**Fig. 2:**
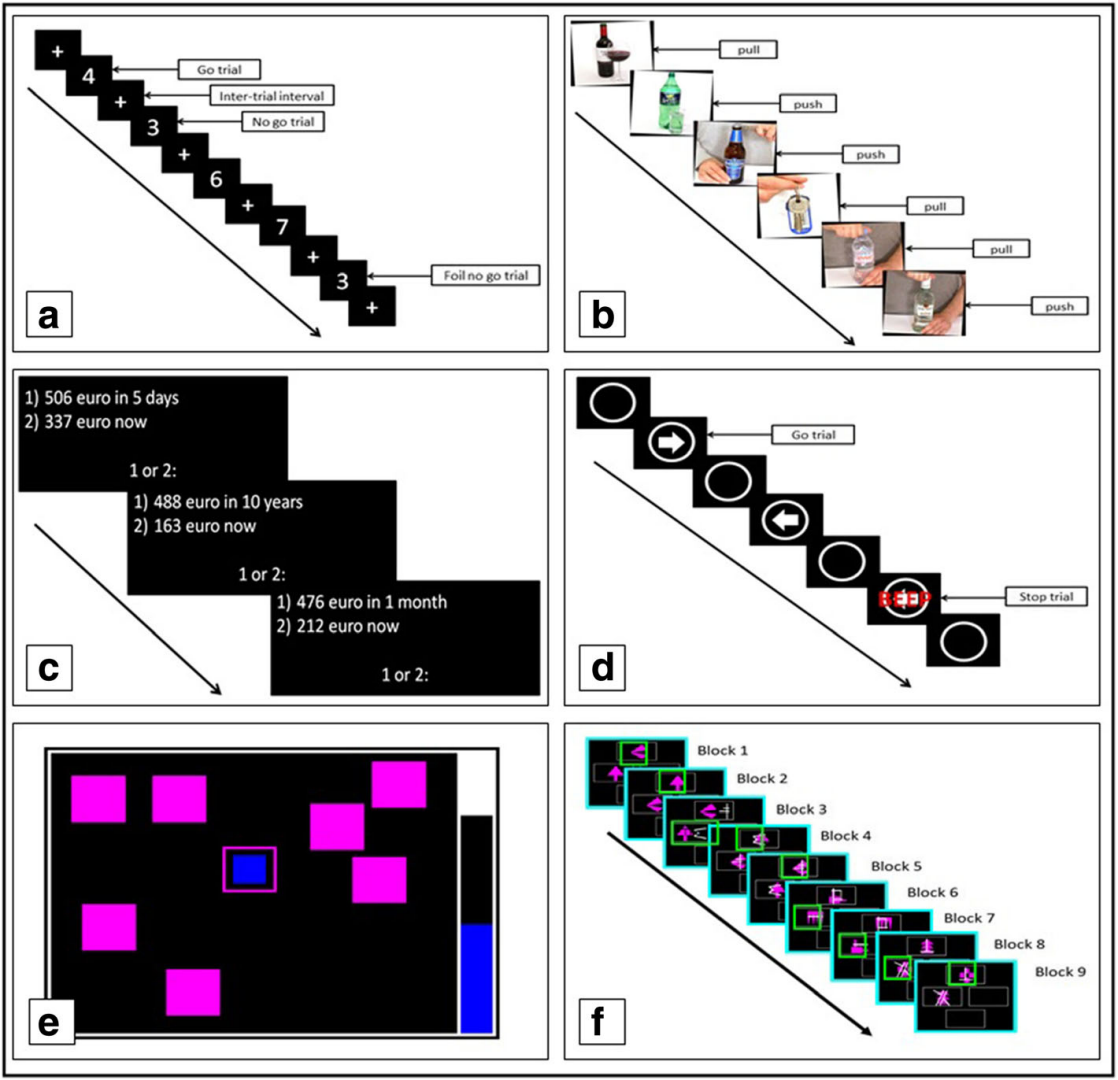
Graphical representation of the neurocognitive measures. **a** Go/ No-go Task (GNGT), **b** Approach Avoidance Task (AAT), **c** Delay discounting task (DDT), **d** Stop signal task (SST), **e** Spatial Working Memory Task (SWMT), **f** Intra-dimensional/ Extra- dimensional Set Shift (IDED). Fig. E-F are adapted from the CANTAB instruction manual

1. Go/ No-go Task (GNGT): This task (adapted from [45]) measures the ability to refrain from action initiation [46]. Participants see a number (‘1’, ‘2’, ‘3’, ‘4’, ‘5’, ‘6’, ‘7’, ‘8’, ‘9’) projected on a screen in front of them. They are instructed to press the spacebar with their right hand as soon as they see a number, but not if it is the number ‘3’ (=no-go trial) (Fig. 2a). The outcome measure will be the number of commission errors, i.e. a response to a no-go trial, reflecting action impulsivity. The higher the number of commission errors, the more impulsive an individual is.
2. Approach Avoidance Task (AAT): This task (adapted from [47]) measures the bias towards approaching alcoholic beverages. During this task pictures from a validated dataset [48] of alcoholic beverages, sodas and neutral objects (for example scissors) are presented on a screen in front of the participant. The pictures are rotated 3° towards the left or right, indicating whether a participant has to pull or push a picture using a joystick (Fig. 2b). When a picture is pulled or pushed the size of the picture increases or decreases respectively. The bias score per stimulus category [alcohol/ soda/ neutral] will be calculated by subtracting the median reaction time of the approach (pull) trials from the median reaction time of the avoid (push) trials. When the result of this subtraction is positive, this indicates a relative faster approach compared to avoid, i.e. an approach bias. When the result of the subtraction is negative this indicates a relatively faster avoid compared to approach, i.e. avoid- bias.
3. Delay Discounting Task (DDT): This task (based on [49]) measures the extent of impulsive decision making. Participants are presented with a choice between an immediate (lower) and a delayed (higher) hypothetical monetary reward (Fig. 2c). The value of the immediate reward varies across the trials in one block, and depends on the responses that are made [49]. The outcome measure will be the area under the discounting curve (AUC), reflecting the degree of discounting by delay [50]. Impulsive choice behavior is indicated by a smaller AUC.
4. Spatial Working Memory Task (SWMT): This task is part of the Cambridge Neuropsychological Test Automated Battery (CANTAB) test battery, and measures the ability of a subject to remember spatial information and manipulate this using working memory. During this task participants are presented with a number of colored squares (or boxes for the participant) shown on a screen. The participant is instructed to find a blue token hidden in each box, and use this token to fill up the black empty space on the right side of the screen (Fig. 2d). In order to open a box, and see whether there is a hidden token inside, the participant needs to touch the box on the screen. If no token is found the participant must continue its search until a token is found. When there is a blue token inside, the participant must now touch the black empty space to fill up this space with the token. Now the participant has to begin a new search. The next token will only be hidden in a box that so far has been empty. This procedure is repeated until all tokens are found and the entire empty space is filled with tokens. The task starts with three boxes, and this will increase to four, six and eight boxes. Touching a box where the token had already been found is considered an error. The outcome measure will be the number of errors a subject makes and reflects working memory capacity. The more errors a subject makes, the lower the working memory capacity.
5. Stop Signal Task (SST): This task (part of the CANTAB test battery) measures the ability of an individual to inhibit an ongoing action [46]. Participants are presented with a white ring on a black screen. In the ring a white arrow pointing either to the left or to the right appears. The participant needs to press the left button if the arrow points to the left and the right button when the arrow points to the right as fast as possible (go trial). During some trials the participant hears an auditory signal (beep), after the arrow appears, which indicates they have to stop their response and not press the button (stop trial) (Fig. 2e). The outcome measure will be the amount of successful stops and reflects the capability of stopping an initiated response. The lower the number of successful stops, the more impulsive an individual is.
6. Intra-dimensional/ Extra- dimensional Set Shift (IDED): This task (part from the CANTAB test battery) tests rule acquisition and reversal learning. Two stimuli are presented on a screen from which the participant has to choose one by pressing on the screen. These stimuli are made up of two artificial dimensions: color-filled shapes and/or white lines. The stimuli presented can be either simple (just one of the two dimensions) or compound (stimuli contain both the dimensions). The task contains 9 blocks increasing in difficulty (Fig. 2f). After pressing the stimulus on the screen the computer gives feedback on whether this was the correct stimulus. In this way participants can learn the task rules. After six correct responses (learning criterion) the program changes the rule, and thereby the participant reaches the next block. If the participant does not reach the learning criterion the test terminates after 50 trials. The outcome measures will be the number of trials needed to reach the next stage (indicating the rule learning capacity), and the number of errors made after a rule change (indicating the capacity of reversal learning) [51]. Higher numbers indicate lower rule learning and reversal learning capacity.

#### Neuroimaging measures

Because the effect of HF- rTMS over the rDLPFC of alcohol dependent individuals on brain functioning is poorly investigated [22], several cognitive tasks will be conducted while functional Magnetic Resonance Imaging (fMRI) is performed. Furthermore, as a control measure, arterial spin labelling (ASL) MRI will be performed in order to investigate changes in cerebral blood flow [52]. MRI scanning will be performed on a 3.0-Tesla Intera full-body scanner (Philips Medical Systems, Best, the Netherlands) with a 32 channel sense head coil located near the Academic Medical Centre in Amsterdam.

1. Cue reactivity task: This task (adapted from [53]) measures the neural response during presentation of alcohol and neutral pictures. The task consists of ten blocks, subdivided into five alcohol and five neutral blocks, presented alternatingly. Each blocks contains seven trials which each show one picture. Of these seven trials, six have a relevant content, and one is a target, namely an animal. The participants are instructed to thoroughly look at all the pictures, and press the right button when they see an animal. Before and after the task the participant will be asked to rate “how much do you feel like drinking alcohol right now?” on a 10 point scale in which 1 indicates “not at all” and 10 indicates “very much” [53]. We will compare neuronal activity during watching alcohol pictures with neuronal activity during watching neutral pictures.
2. Stroop task: This task (adapted from [54]) measures the neural response during watching two types of stimuli, congruent or incongruent. All trials contain one of the following words in Dutch: “red”, “blue”, “yellow”, “green”. In congruent trials the color of the word is the same as the content of the word, while during incongruent trials the color of the word is different from the content of the word. Participants are instructed to indicate the color of the word by pressing on the representative button [54]. We will compare neuronal activity during the incongruent trials with the congruent trials.
3. Monetary Incentive Delay Task (MIDT): This task adapted from [55, 56] measures the neural response during the anticipation of a reward. Participants are presented with cues (blue triangle or blue circle) that indicate whether they could earn €0.01 or €0.50. The cue is followed by a target (green star). The participant is instructed to press the right button as soon as possible when they see the target. If the participant is fast enough, he/she earns the amount of money. This is communicated to the participant through a feedback screen in which both the amount of earned money as well as the total amount of money is presented. We will compare the neuronal response during the anticipation of the high reward with activity during the anticipation of the low reward.
4. Resting state task: During this scan (adopted from [57]) the neuronal activity of the resting state network will be measured. Participants are presented with a black screen and instructed to close their eyes, not think of something in particular, just let their minds wander and try not to fall asleep [23]. Functional connectivity of the rDLPFC will be determined.

### Statistical analyses

#### Power analysis

The current study is the first to investigate the effect of multiple HF-rTMS sessions on abstinence rates measured six months after the last stimulation session. To the best of our knowledge no studies thus far have investigated the effect of rTMS on abstinence after a longer - clinically more relevant - period of time. Therefore no effect size is known or can be calculated, which makes it impossible to perform a scientifically correct power analysis. However, to estimate how many participants we would need, we based our population estimate on a previous study with a similar design [20], which used craving as the outcome of interest. Although our secondary outcome measure is craving, we hypothesized that we should at least include a similar number of participants (30 participants in the active stimulation group) as [20]. Given a drop-out percentage of 10%, and an estimated lower effect on behavioral measures compared to craving, the current study will include 38–40 alcohol dependent individuals per group, resulting in a total of approximately 80 participants.

##### Neuroimaging study part

For the neuroimaging sub-study no information about the effect-size is available. However, studies of the statistical properties of one large fMRI cohort found that the sensitivity and reproducibility of group analyses reaches a plateau at *N* = 27 [58]. With this sample size, the proportion of correct classification of truly active and inactive voxels corrected for chance is k > 0.75. In line, this sample size is also similar to the sample size that is typically used for neuroimaging studies that compare psychiatric patients with healthy controls (*N* = 20–30) [59]. Therefore 28 participants of each group will be included in the neuroimaging study part.

#### Descriptive statistics

Descriptive analyses will be performed in order to see whether randomization resulted in two research groups with a similar distribution of demographic factors. Appropriate parametric and non-parametric statistical tests will be used to analyze descriptive statistics, and if required, multiple comparison corrections will be performed.

#### Primary outcome measure

Data of the primary outcome measure will be analyzed in accordance with the intention-to-treat principle. Missing data of subjects that are randomized and received at least one stimulation session will be imputed in order to achieve complete datasets. Subsequently an appropriate test for comparing two groups will be used depending on the distribution of the outcome measure. Additionally, data of the primary outcome measure will be analyzed with only treatment completers (according to the per protocol analysis), again using an appropriate test depending on the distribution of the outcome measure.

#### Secondary outcome measures

An appropriate test for comparison of two groups will be used depending on the distribution of the outcome measures ‘days until first relapse’ and ‘total alcohol consumption’.

Multilevel models (capable of handling missing data points) with predictors time, treatment and time X treatment interaction will be used to test for the effect of the HF-rTMS add-on treatment on craving. The following time points will be included: post HF-rTMS, 3 months follow up and 6 months follow up.

#### Additional clinical outcome measures

##### Explorative alcohol use parameters

An appropriate test for comparison of two groups will be used depending on the distribution of the outcome measure ‘full abstinence rate’. An appropriate test for comparing a categorical variable between two groups will be used for the outcome measure ‘treatment success’.

##### Additional craving assessment

Multilevel models (capable of handling missing data points) with predictors time, treatment and time X treatment interaction will be used to test for the effect of the HF-rTMS add-on treatment on craving measured with the OCDS. The following time points will be included: post HF-rTMS, 3 months follow up and 6 months follow up. Data of the VAS scales will be analyzed by calculating difference scores between pre and post HF-rTMS. These scores will be used in a multilevel model (capable of handling missing data points) with predictors time, treatment and time X treatment interaction to test for the effect of the HF-rTMS add-on treatment.

##### Questionnaires

Multilevel models (capable of handling missing data points) with predictors time, treatment and time X treatment interaction will be used to test for the effect of the HF-rTMS add-on treatment on questionnaire scores. Total scores of baseline, intermediate and post-rTMS data will be compared.

##### Supplementary measures

These measures will be compared between groups using an appropriate statistical test depending on the distribution of the outcome measure.

#### Neurocognitive measures

Multilevel models (capable of handling missing data points) with predictors time, treatment and time X treatment interaction will be used to test for the effect of the HF-rTMS add-on treatment on neurocognitive measures. The following time points will be included: baseline, intermediate and post HF-rTMS.

#### Neuroimaging measures

The software package Statistical Parametric Mapping (SPM) (Wellcome Department of Cognitive Neurology, London, UK) will be used to analyze the task induced (cue reactivity, stroop, MIDT) activation patterns. First, images will be preprocessed, including motion correction, normalization to correct for individual differences in anatomy and smoothing. Thereafter, individual subject analyses will be performed within the context of the General Linear Model (GLM), using delta functions convolved with a synthetic hemodynamic response function to model events of interest. Contrast images containing parameter estimates for each comparison of interest will be entered into second level analyses to assess baseline versus HF-rTMS effects. For the resting state activation spatiotemporal independent component analysis will be employed.

ASL scans will be analyzed using the Explore ASL toolbox in Matlab. First, the T1 images are normalized and segmented into grey and white matter. The probability maps and the gray matter tissue probability maps are then spatially normalized using the Diffeomorphic Anatomical Registration analysis using Exponentiated Lie algebra (DARTEL) algorithm [60]. Then, for the ASL time series, motion estimation is performed, as well as exclusion of frames with motion spikes. Subsequently, label and control images are subtracted and corrected for slice gradients. After this, the perfusion weighted images are registered to the gray matter tissue probability maps of each subject using 6 parameter rigid body registrations, followed by voxel-based outlier rejection. Then, cerebral blood flow images will be quantified with the following parameters: post-labeling delay = 1525 ms, T1_arterial_ = 1650 ms, labeling efficiency α = 0.8, labeling duration τ = 1650 ms [61]. Finally, all transformations will be mathematically combined in a single B-spline interpolation and applied to the CBF maps. This results in an average perfusion image per participant which will be used for the assessment of baseline versus HF- rTMS effects.

## Discussion

This paper presents a single blind randomized clinical trial protocol investigating whether 10 sessions of active HF-rTMS compared with 10 sessions of sham HF-rTMS improve the treatment outcomes of alcohol dependence. The aim of this study is to increase abstinence rates, decrease craving, and improve neurocognitive and brain functioning measures relevant for alcohol dependence treatment.

The main challenge of this study will be completion of the entire follow-up procedure. Participants will be called three, six and twelve months after finishing the last stimulation session to assess the number of abstinent days. Within the population of alcohol dependent individuals, chances are high that participants will get lost at follow-up [62]. There is a risk that we may not get the primary outcome measure for all participants, because it is uncertain whether we can reach all participants at six months follow-up. However, our research group is experienced with a six months follow-up and no main problems have been reported in reaching participants.

The first strength of this study is that it investigates the effect of *multiple* HF-rTMS session. The second strength of this study is that it takes into account several aspects that are important in the treatment of alcohol dependence. So far studies mainly looked at the effect of HF-rTMS on self-reported craving, although the most relevant clinical question is whether HF-rTMS has an effect on abstinence [22]. This study will perform follow-up measurements to assess the number of abstinent days in the six months after the last neurostimulation session. Furthermore this study will elucidate the underlying mechanism by which rTMS may induce its effects in alcohol dependent individuals by investigating several neurocognitive as well as neuroimaging measures [22].

The limitation of this study is that the trial is not double blind controlled. However, a double blind controlled rTMS study with a sham condition similar to this study is impossible because the researcher needs to tilt the coil 90° relative to the skull [23]. Because this study only uses participants self-report measures and the researcher does not score any clinical effects, the outcome measures are not affected by the knowledge of the researcher, and a double blind paradigm is not necessary.

If this study reveals higher abstinence rates and decreased craving in the active stimulation group compared with the sham stimulation group, and the active HF-rTMS induces negligible side effects, this may lead to larger clinical trials. If most of these trials find positive results of multiple HF-rTMS sessions on treatment outcomes, this eventually could result in approval by the regulatory authorities as additional treatment method for substance dependence, just as for instance the Food and Drug Administration has approved HF-rTMS for the treatment of depression.

## Abbreviations

AAT: Approach avoidance task
ASL: Arterial Spin Labeling
AUDIT: Alcohol use disorders identification test
AUQ: Alcohol urge questionnaire
BDI: Beck depression inventory
BIS: Behavioral inhibition system
BIS/BAS: Behavioral inhibition and behavioral activation systems
CANTAB: Cambridge neuropsychological test automated battery
CBT: Cognitive behavioral therapy
DDT: Delay discounting task
DLPFC: Dorsolateral prefrontal cortex
DSM: Diagnostic and statistical manual of mental disorders
fMRI: Functional magnetic resonance imaging
GNGT: Go/ No-go task
HF: High frequency
IDED: Intra-dimensional/ extra- dimensional Set Shift
MATE: Measurement in the addictions for triage and evaluation
MIDT: Monetary incentive delay task
MINI: Mini international neuropsychiatric interview
MOCA: Montreal cognitive assessment
MRI: Magnetic resonance imaging
NLV: Dutch version of the adult reading test
NTR: Netherlands national trial register
OCDS: Obsessive compulsive drinking scale
PANAS: Positive and negative affect scale
rDLPFC: Right dorsolateral prefrontal cortex
rTMS: Repetitive transcranial magnetic stimulation
SPM8: Statistical parametric mapping 8
SPSS: Statistical package for the social sciences
SST: Stop signal task
STAI-STATE: State trait anxiety inventory
SWMT: Spatial working memory task
TAU: Treatment as usual
TLFB: Time line follow back
UPPS: Urgency, premeditation, perseverance, sensation seeking
VAS: Visual analogue scale
WAIS: Wechsler adult intelligence scale
